# Prevalence, pattern and risk factors of retinal vein occlusion in an elderly population in Nepal: the Bhaktapur retina study

**DOI:** 10.1186/s12886-017-0552-x

**Published:** 2017-09-02

**Authors:** Raba Thapa, Sanyam Bajimaya, Govinda Paudyal, Shankar Khanal, Stevie Tan, Suman S. Thapa, Ger van Rens

**Affiliations:** 1 Tilganga Institute of Ophthalmology, Kathmandu, Nepal; 2 Central Department of Statistics, Tribhuvan University, Kirtipur, Nepal; 3 Vrije University Medical Center, Amsterdam, The Netherlands; 4 Vitreo-retina Service, Tilganga Institute of Ophthalmology, P O Box, Kathmandu, 561 Nepal

**Keywords:** Prevalence, Risk factors, Retinal vein occlusion, Branch retinal vein occlusion, Central retinal vein occlusion, Nepal

## Abstract

**Background:**

This study aims to explore the prevalence, pattern and risk factors of retinal vein occlusion (RVO) in an elderly population of Nepal.

**Method:**

One thousand eight hundred sixty subjects of age 60 years and above were enrolled in a population-based, cross-sectional study. Detailed history, visual acuity, anterior segment and posterior segment examinations were done. Blood pressure, non-fasting blood sugar, body mass index and abdominal girth were measured. Retinal vein occlusions were further divided into branch retinal (BRVO), hemi-retinal and central retinal vein occlusion (CRVO).

**Result:**

Age ranged from 60 to 95 years with a mean of 69.64 ± 7.31 years. Overall population prevalence for RVO was 2.95% (95% Confidence interval (CI): 2.23–3.83), BRVO 2.74% (95% CI: 2.05–3.58) and CRVO 0.21% (95% CI: 0.06–0.55). BRVO was seen in 51 subjects (92.73%) and CRVO in 4 (7.27%). Among the total RVO, unilateral and bilateral involvement was 85.45% and 14.55%, respectively. Among the subjects with BRVO and CRVO, 37.25% and 50% had low vision, respectively. The risk of RVO increased with ageing and was more among males. There was an increased risk of RVO among those with hypertension, and with diabetes and hypertension. There was also an increased risk of RVO among subjects with hypermetropia, those with pseudophakia and those who were smokers and consumed alcohol.

**Conclusion:**

Retinal vein occlusion is a common retinal vascular disorder in the elderly population of Nepal. The main risk factors for RVO were increasing age and hypertension.

## Background

Retinal diseases are an emerging cause of visual impairment and blindness in the world where retinal vein occlusions (RVO) is the second most common retinal vascular diseases after diabetic retinopathy [1–3]. Changes in life style habits which may lead to increase in systemic diseases such as hypertension, diabetes mellitus, cardiovascular diseases and hyperlipidaemia predispose to retinal problems [4–8]. Untimely detection and delayed treatment can result in irreversible visual impairment and blindness from vision threatening sequelae [9–12].

Several studies conducted in the Caucasian population aged from 40 years and above have shown that the prevalence of branch retinal vein occlusion (BRVO) and central retinal vein occlusion (CRVO) ranges between 0.6% - 1.1% and 0.1–0.4%, respectively [13–15]. In the few Asian studies, the prevalence of BRVO and CRVO was 0.6% and 0.2% in those more than 40 years of age [16, 17]. RVO was the most common retinal vascular disease in a population based study of Nepal [18]. This is the first population-based study to estimate the pattern and risk factors for RVO in Nepal.

## Methods

### Study population

Bhaktapur district has 2 municipalities and 161 village development committees. According to the 2001 census the population of Bhaktapur was 298,704 and 48,223 were above the age of 40 years. In the Bhaktapur Glaucoma Study (BGS) conducted from 2007 to 2010, a WHO 30 cluster sampling method was used. From these 30 clusters, a house to house enumeration was done and a name list prepared. From this name list 4800 subjects above the age of 40 years were selected using a EPI-INFO software, version 3.5.1 [19]. From these 4800 subjects only those above 60 years of age were re-invited for an examination. The total sample size of the Bhaktapur Retina Study (BRS) was 2100 of which 62% is the surviving sample of the BGS. From the remaining subjects above the age of 60 years, 15% of the subjects had passed away, 5% had moved to other places while 18% of the subjects were unable to visit the study site. In order to meet the calculated sample size of 2100, the rest 38% were selected from the adjoining clusters as a cross sectional survey. Two female community health workers visited the subjects at their homes and invited them to participate in the study. The required sample size for this study was estimated to be 2100 subjects after assuming 7% prevalence for vitreo-retinal disorders in individuals 60 years and older, a relative precision of 25%, 85% compliance, and a design effect of 2. The 7% prevalence of vitreo-retinal disorder was derived from the occurrence of retinal disorders in the BGS [18]; however, this analysis has been carried out with 1860 subjects (88.57%) with complete information after excluding other subjects with incomplete information. The prevalence and risk factors of RVO were assessed in this population. All subjects were referred to the primary eye care centre of Bhaktapur district for a detailed evaluation. The study was conducted from August 2013 to December 2015.

A structured questionnaire was developed to assess the prevalence and risk factors for RVO. Mid-level ophthalmic personnel were involved in the interview, and two ophthalmologists were involved in examination of study subjects. 50 cases were pre-tested. No respondents reported difficulties in answering the questionnaire, and there were no statistically significant variations in examination findings. When the subjects were able to read and write in the national language, they were categorized as literate as defined by the Government of Nepal. The predominant profession was considered as the occupation.

### Patient examination and retinal vein occlusion assessment

All patients provided a detailed ocular and medical history, followed by anterior segment and dilated fundus examinations including measurement of intraocular pressure. The best corrected visual acuity (BCVA) was assessed using the logarithm of minimum angle of resolution (log MAR) with tumbling E charts placed at 4 m. Two retina specialists performed standardized eye examinations on the patients. A total of five fundus photographs were taken of each eye after mydriasis using a digital fundus camera (Canon) by a trained mid-level ophthalmic technician who had been government certified course to provide primary eye care in ophthalmology.

Visual impairment was based on the International Classification of Disease 10th edition [20]. Briefly, visual impairment was defined as visual acuity (VA) less than 6/18 (<20/60; <0.3 Log MAR) in the better eye with best correction. Low vision was defined as a BCVA of less than 6/18 (<20/60; <0.3 Log MAR) but not less than 3/60 (20/400; 1.3 Log MAR) in the better eye. A VA of less than 3/60 (<20/200; <1.3 Log MAR) with best correction in the better eye was considered blindness.

RVO was grouped into central retinal vein occlusion, hemi-central retinal vein occlusion (Hemi-CRVO) and branch retinal vein occlusion. Briefly, in BRVO, there is occlusion of a branch of a retinal vein system and is further divided into major BRVO and macular BRVO. In CRVO, the occlusion is located in the central retinal vein and is further divided into ischemic and non-ischemic types. The hemi CRVO, where there is involvement of only one half of retina surface was also divided in to ischemic and non-ischemic types [21, 22].

### Assessment and definitions of risk factors

A detailed history was obtained using a standardized questionnaire. All subjects underwent blood examination for non-fasting blood sugar levels, and measurements of blood pressure, height, weight, and abdominal girth were recorded using standard techniques. Age, gender, education status, occupation, smoking, alcohol consumption, sunlight exposure, consumption of antioxidant vitamins, and presence of systemic problems like diabetes mellitus, hypertension, hyperlipidaemia, and other cardiac disorders were elicited from the self-reported history. Body mass index (BMI) was calculated by the formula; Body Mass Index = weight in kilograms divided by the square of the height in meters (Kg/m^2^). Venous blood samples were taken for assessment of non-fasting blood sugar. The diagnosis of diabetes mellitus was based on either the use of diabetic medications or a random blood sugar level of 200 mg/dl or greater [18, 23]. Blood pressure (BP) was measured on all subjects who were categorized as hypertensive if systolic blood pressure was 140 mmHg or more, diastolic blood pressure was 90 mmHg or more, or if they used antihypertensive medications.

The study was approved by the Institutional Review Board and Ethics Committee of Tilganga Institute of Ophthalmology (TIO) and conducted in accordance with the Declaration of Helsinki. Informed consent was written in the vernacular and was read out for those unable to read. Subjects were asked to sign the consent form, and thumb impressions were taken for those unable to sign prior to enrollment in the study.

### Statistical analysis

Summary measures such as mean ± sd for continuous variables and percentages for categorical variables were computed. Comparisons of continuous variables between two groups were analyzed by using independent t- tests, and the associations between two categorical variables were assessed using Chi-square or Fisher’s exact test wherever applicable. Presence of retinal vein occlusion was considered as a binary outcome variable. In order to quantify the effect of different risk factors on the outcome variable, they were measured thorough the use of logistic regression. The prevalence of CRVO, BRVO, etc. was computed with 95% confidence intervals. Results were considered significant at 5% level of significance (*p* value ≤0.05). All of the statistical analyses were performed using STATA 9.0.

## Results

Complete information was available for 1860 subjects (88.57%). The non-responder and responder groups were compared with regard to age and gender. There was no significant difference observed between age and gender among the two groups (Table 1).

**Table 1:** Comparison of responders and non responders in the study population

Variable	Responders (*n* = 1860)	Non responders (*n* = 240)	*p*- value
Mean age (Years)	69.64 ± 7.31	69.50 ± 7.93	0.782
Male, n (%)	821 (44.14)	110 (45.83)	0.629
Female, n (%)	1039 (55.86)	130 (54.17)	

The characteristics of the study population in various stages of RVO are shown in Table 2. The age ranged from 60 to 95 years with a mean age of 69.64 ± 7.31 years. Half of the subjects (51.08%) were between 60 and 69 years of age, whereas 11.45% were 80 years and above. More females 1039 (55.86%) participated in the study. Among the total, 1433 (77.04%) were illiterates, and 1351 (72.63%) were farmers by occupation.

**Table 2:** Demographic characteristics among subjects with retinal vein occlusion

Demographic characteristics	All Persons (N/%) (*N* = 1860)	RVO Total (N/%) (*N* = 55)	BRVO (N/%)(*N* = 51)	CRVO (N/%) (*N* = 4)	*p*- value
Age(years)					
60–69	950 (51.08)	27 (49.09)	24 (47.06)	3 (75)	0.521
70–79	697 (37.47)	22 (40)	21 (41.18)	1 (25)	
≥ 80	213 (11.45)	6 (10.91)	6 (11.76)	0	
Gender					
Male	821 (44.14)	25(45.45)	24(47.06)	1 (25)	0.394
Female	1039 (55.86)	30 (54.55)	27 (52.94)	3 (75)	
Occupation					
Agriculture	1351 (72.63)	40 (72.73)	37 (72.55)	3 (75)	0.916
Other occupations	509 (27.37)	15 (27.27)	14 (27.45)	1 (25)	
Education status					
Illiterates	1433 (77.04)	48 (87.27)	44 (86.27)	4 (100)	0.428
Literates	427 (22.96)	7 (12.73)	7 (13.73)	0	

*RVO* Retinal vein occlusion, *BRVO* Branch retinal vein occlusion, *CRVO* Central retinal vein occlusion

RVO was found in 55 subjects in the study population with an overall population prevalence of 2.95% (95% Confidence interval (CI); 2.23–3.83). BRVO was found in 51 subjects (92.73%) and CRVO in 4 subjects (7.27%) among the RVO subjects. The population prevalence of BRVO was 2.74% (95% CI: 2.05–3.58) and CRVO 0.21% (95% CI: 0.06–0.55). Among the total number of RVO subjects, unilateral involvement was documented in 47 subjects (85.45%), while involvement was bilateral in 8 subjects (14.55%).

Table 2 shows the demographic characteristics of the persons with RVO. Almost half (49.09%) of the total number of subjects with RVO was within the age range of 60–69 years. Similarly, among the total BRVO and CRVO subjects, both BRVO (47.06%) and CRVO (75%) were higher in the age group 60–69 years.

RVO, BRVO and CRVO each was seen more common among females, agricultural occupation, and illiterates. However, this was not statistically significant amongst the groups.

Among the study subjects, hypertension was found in 45.45% in RVO, 56.07% in BRVO and 25% in CRVO. Only 7.27% of subjects with RVO had diabetes mellitus, and all of them had BRVO. RVO was found in 5.45% of the subjects having both diabetes and hypertension, and all CRVO patients had both diabetes and hypertension. RVO was also higher (60.78%) among the subjects with BMI less than 24.9 Kg/m^2^. The mean abdominal girth was higher among the subjects with BRVO (78.53%) than in CRVO (76.50%). 63.64% of RVO subjects had BCVA less than 0.3 Log MAR. Myopia and hypermetropia was found in 10.91% and 3.45% of RVOs respectively, while the rest of the subjects had mixed types of refractive error and astigmatism. None of the subjects with CRVO had hypermetropia. Pseudophakia was found in 21.82% of RVO subjects, and 50% of the subjects with CRVO had pseudophakia. 60% of those with RVO were smokers, 60.78% of subjects with BRVO, and 50% of the subjects with CRVO were smokers. 41.82% of the subjects with RVO were alcohol consumers (Table 3).

**Table 3:** Systemic, ocular conditions and social habits among the subjects with retinal vein occlusion

Systemic Conditions	RVO Total (N, %)	BRVO (N, %)	CRVO (N, %)	*p*- value
Hypertension				
No Hypertension	30 (54.55)	27 (52.94)	3 (75)	0.394
Hypertension	25 (45.45)	24 (47.06)	1 (25)	
Diabetes Mellitus				
No diabetes mellitus	51 (92.73)	47 (92.16)	4 (100)	0.561
Diabetes mellitus	4 (7.27)	4 (7.84)	0	
Combined diabetes and hypertension				
No diabetes and hypertension	52 (94.55)	48 (94.11)	4 (100)	0.618
Diabetes and hypertension	3 (5.45)	3 (5.89)	0	
BMI(Kg/m^**2**^)				
≤ 24.9	34 (61.82)	31 (60.78)	3 (75)	0.573
≥ 25	21 (38.18)	20 (39.22)	1 (25)	
Abdominal girth (mean in cm)	78.38 ± 11.09	78.53 ± 9.49	76.5 ± 10.75	0.408
Ocular Factors				
BCVA(Log MAR)				
< 0.3	35 (63.64)	33 (64.71)	2 (50)	0.556
> 0.3	20 (36.36)	18 (35.29)	2 (50)	
Refractive error				
Myopia	6 (10.91)	5 (9.80)	1 (25)	0.589
Hypermetropia	3 (5.45)	3 (5.88)	0	
Astigmatism	46 (83.64)	43 (84.31)	3 (75)	
Lenticular status				
No pseudophakia	43 (78.18)	41 (80.39)	2 (50)	0.156
Pseudophakia	12 (21.82)	10 (19.61)	2 (50)	
Social habits				
Smoking				
Never	22 (40)	20 (39.22)	2 (50)	0.913
Present	17 (30.91)	16 (31.37)	1 (25)	
Past	16 (29.09)	15 (29.41)	1 (25)	
Alcohol consumption				
Never	22 (40)	20 (39.22)	2 (50)	0.775
Present	23 (41.82)	22 (43.14)	1 (25)	
Past	10 (18.18)	9 (17.65)	1 (25)	

*RVO* Retinal vein occlusion, *BRVO* Branch retinal vein occlusion, *CRVO* Central retinal vein occlusion, *BCVA* Best corrected visual acuity

38.18% of RVO subjects had low vision in better eyes in best correction. Among the subjects with BRVO and CRVO, low vision was found in 37.25% and 50%, respectively, but the difference was not statistically significant (*p* = 0.613) (Table 4).

**Table 4:** Best Corrected Visual acuity among the subjects with RVO

Visual Acuity	Total RVO	BRVO (N, %)	CRVO (N,%)
Normal visual acuity	34 (61.82)	32 (62.75)	2 (50)
Low vision	21 (38.18)	19 (37.25)	2 (50)
Total	55 (100)	52 (100)	4 (100)

*RVO* Retinal vein occlusion, *BRVO* Branch retinal vein occlusion, *CRVO* Central retinal vein occlusion

There was a slight increase in the risk of developing RVO in the 70–79 year age group when compared to other age groups; however, this was not statistically significant. Males, agricultural occupation, and illiterates also had a higher risk of RVO but this was not statistically significant.

There was increased risk of RVO among those with hypertension, and those having both diabetes and hypertension. The risk was lower, however, among the subjects who had only diabetes. High body mass index and abdominal girth were not associated with RVO. Similarly, there was an increased risk of RVO among the subjects with hypermetropia and pseudophakia, although this was not statistically significant. The risk of RVO was also high among those who consumed alcohol and tobacco. No variable was a significant risk factor for retinal vein occlusion when we performed univariate logistic regression analysis (Table 5).

**Table 5:** Risk factors for retinal vein occlusion using logistic regression analysis

Characters	No RVO (*n* = 1805) Number (%)	RVO (*n* = 55) Number (%)	Odds Ratio (95% Confidence Interval)	*P* - value
Age in Years, n (%):				
60–69	923 (51.14)	27 (49.09)	1.00	
70–79	675 (37.40)	22 (40.00)	1.11(0.62–1.97)	0.711
≥ 80	207 (11.47)	6 (10.91)	0.99(0.40–2.43)	0.984
Gender:				
Male, n(%)	796 (44.10)	25 (45.45)	1.00	
Female, n(%)	1009 (55.90)	30 (54.55)	0.94 (0.55–1.62)	0.842
Occupation:				
Agriculture	1311 (72.63)	40 (72.73)	1.00	0.987
other occupations	494 (27.37)	15 (27.17)	0.99 (0.54–1.82)	
Literacy:				
Illiterate	1385 (76.73)	48 (87.27)	1.00	
Literates	420 (23.27)	7 (12.73)	0.48 (0.21–1.07)	0.073
All Hypertension				
No	1187 (65.16)	30 (54.55)	1.00	
Yes	618 (34.24)	25 (45.45)	1.60 (0.93–2.74)	0.088
Known Hypertensive				
No	1401 (72.62)	36 (65.45)	1	
Yes	404 (22.38)	19 (34.55)	1.83 (1.03–3.22)	0.037
Newly diagnosed Hypertensive				
No	1587 (87.92)	49 (89.09)	1	
Yes	218 (12.08)	6 (10.91)	0.89 (0.37–2.10)	0.793
Diabetes				
No	1641 (90.91)	51 (92.73)	1.00	
Yes	164 (9.09)	4 (7.27)	0.78 (0.28–2.18)	0.645
Known diabetes				
No	1672 (92.63)	51 (92.73)	1	
Yes	133 (7.37)	4 (7.27)	0.98 (0.35–2.76)	0.979
Newly diagnosed diabetes	1774 (98.28)	55 (100)		
	31 (1.72)	0		0.327
Combined HTN and Diabetes mellitus				
No	1710 (94.74)	52 (94.55)	1.00	
Yes	95 (5.26)	3 (5.45)	1.03 (0.31–3.38)	0.950
Body Mass Index (kg/m^**2**^)				
(< 24.9)	1103 (61.11)	34 (61.82)	1.00	
(>25)	702 (38.89)	21 (38.18)	0.97 (0.55–1.68)	0.915
Body Mass Index(kg/m^**2**^) (mean ± S.D)	23.66 ± 4.00	23.41 ± 4.58	0.98 (0.92–1.05)	0.654
Abdominal girth (mean ± S.D)	80.29 ± 11.13	78.38 ± 9.49	0.98 (0.95–1.00)	0.203
BCVA (Log MAR)				
< 0.3	1139 (63.10)	35 (63.64)	1.00	0.936
> 0.3	666 (36.90)	20 (36.36)	0.97 (0.55–1.70)	
Myopia	181 (10.03)	6 (10.91)	1.00	
Hypermetropia	246 (13.63)	3 (5.45)	1.17 (0.75–1.85)	0.215
Others	1378 (76.34)	46 (83.64)	1.00 (0.42–2.39)	0.987
Pseudophakia				
No	1526 (85.10)	43 (78.18)	1.00	0.162
Yes	269 (14.90)	12 (21.82)	1.59 (0.82–3.06)	
Smoking				
Non smoker	790 (43.74)	22 (40)	1.00	0.582
Smoker	1016 (56.26)	33 (60)	1.16 (0.67–2.01)	
Alcohol				
No Alcohol	772 (42.20)	22 (40)	1.00	0.685
Alcohol	1034 (57.25)	33 (60)	1.11 (0.64–1.93)	

*RVO* Retinal vein occlusion, *HTN* Hypertension, *BCVA* Best corrected visual acuity

## Discussion

This is the first population-based study to estimate the pattern and risk factors of retinal vein occlusion in Nepal. The outcomes of the study can help apply measures in the community to reduce blinding sequelae of RVO.

The overall population prevalence of RVO in this study was 2.95%. This was slightly higher than other population based studies conducted in the developed and developing countries that ranged from 0.6 to 1.6% [13, 15, 16, 24, 25]. One possible reason for the higher prevalence could be due to the more elderly age of our participants. Our study may be compared with a another conducted by Mitchell et al. in Australia [12] where the prevalence of RVO was 1.2% for the age group 60–69 years, 2.1% for the age group 70–79 years and 4.6% for the age group 80 years or older. In our study, the prevalence of RVO was 2.84% for the age group 60–69 years, 3.15% for the age group 70–79 years, and 2.82% for the age group 80 years and above. The prevalence of RVO was slightly higher in the younger age group in this study as compared to the Mitchell et al. however; the prevalence of RVO at the age group 80 years and above was lower in our study. In our study, the mean age of the study participants was 69.64 ± 7.31 years and study was conducted at the age 60 years and above. The study participants included 49 years and above in the series of Mitchell et al. The possible reason could be due to the variation in sample size between the two studies (3654 in Mitchell et al. whereas 1860 in this study).

The population prevalence of BRVO was 2.74%, which was slightly higher than studies conducted in the Caucasian and Asian races which ranged from 0.6–1.1% [14–17]. This difference again could be attributed to the more elderly people in our study. BRVO was more common than CRVO in this study, comprising 92.73% of total RVO, 12.75 times more frequent than CRVO, which is similar to other studies [1, 6, 7, 12]. BRVO is commonly associated with systemic hypertension [8, 25, 26]. Systemic hypertension occurs more frequently in the elderly population [27] which could be the reason for a higher prevalence of BRVO in our elderly population [8]. Since our sample did not estimate CRVO in a younger age group (< 60 years) where it is seen more frequently [11, 29]. We could have also underestimated the prevalence of CRVO. The population prevalence of CRVO was 0.21%. The finding was consistent with other studies where prevalence ranged from 0.1–0.4% [13, 16, 17].

There was bilateral involvement in 14.55% with RVO in our study. Mitchell et al. [12] reported bilateral involvement in 5.1% of subjects. The higher percentage in our population could be due to more elderly population and also uncontrolled systemic diseases. The glycosylated hemoglobin and lipid panel would be helpful for further analysis. The difference in the laterality may be due to variation in sample size between the two study.

Bilateral involvement is a serious public health issue which can lead to visual impairment.

BRVO and CRVO rates were high among subjects with hypertension, which is consistent with other studies [6–8, 12, 24, 26, 28]. Among the subjects with RVO, four were diagnosed with diabetes mellitus and all were BRVO, which implies that DM could be a risk factor for BRVO. Diabetes mellitus has been found to be a risk factor for CRVO [8, 24, 26]. However, due the low number of subjects with CRVO in our study, we are unable to comment further on diabetes as a risk factor in this population.

There are several studies, which have found hypermetropia as a risk factor for CRVO [6, 29]. However, in our study none of the subjects with CRVO had hypermetropia, and thus we are unable to comment on the role of hypermetropia as a risk factor for CRVO, but hypermetropia was seen more frequently in those with BRVO, which is similar to other studies [30]. Almost one third subjects with BRVO and half of the subjects with CRVO had poor visual acuity. Our finding was similar to the series by Mitchell et al. [12].

The risk of RVO was higher with increasing age [12, 24]. RVO was more commonly seen in males, but gender did not prove to be a significant risk factor [1, 25, 26]. We are unable to correlate occupation and literacy as risk factors for RVO because the large majority of the sample population belonged to an agricultural occupation and were illiterate.

The risk of RVO was higher among those with hypertension in this study. This finding was consistent with other hospital-based and population-based studies across the globe [6, 7, 12, 15, 26, 28]. Similarly, combined diabetes and hypertension was associated with an increase in risk of developing RVO [6, 7, 26]. The risk of RVO was low among subjects with diabetes unlike other studies where diabetes was a risk factor of RVO [7, 8, 26]. The risk of RVO was high among those with hypermetropia as compared to myopia in this study. This finding was consistent with other hospital-based and population-based studies from both the developed and developing world [6, 30]. The higher risk of RVO among the subjects with pseudophakia in this study may be associated with an increase in age.

The risk of RVO was higher among the subjects who consumed alcohol and those who were smokers, similar to the series by Klein et al. [13]. The prevalence of RVO diseases is mostly related to systemic diseases such as HTN and DM. The prevalence of RVO can decrease by reducing the rates of DM and HTN and keeping them under control. Screening of high-risk subjects for early detection and timely treatment will reduce the blinding sequelae from RVO [2, 4–32].

The strength of the study is the large sample size of the elderly age group. The major limitation of the study is that we are not able to comment on the prevalence and risk factors of RVO in the younger segment of the study population. Other limitations are that glycosylated haemoglobin and lipid panel tests were not possible to conduct in this population based study for further exploration of risk factors.

## Conclusion

Retinal vein occlusion is a common retinal vascular disorder in the elderly population of Nepal. The main risk factors of RVO were increasing age and hypertension. Regular eye examinations in the high-risk group coupled with timely detection and treatment of retinal vascular occlusions could help prevent blindness in this elderly age population.

## Abbreviations

BCVA: Best corrected visual acuity
BGS: Bhaktapur Glaucoma Study
BMI: Body mass index
BP: Blood pressure
CI: Confidence interval
CRVO: Central retinal vein occlusion
DM: Diabetes mellitus
Hemi CRVO: hemi central retinal vein occlusion
HTN: Hypertension
LogMAR: Logathirm of Minimum Angle of Resolution
N: Number
N: Number
RVO: Retinal vein occlusion
S.D.: Standard deviation
TIO: Tilganga Institute of Ophthalmology
